# Awareness and perception of antimicrobial stewardship program among healthcare professionals in the hospitals of ministry of health and population, Egypt: antimicrobial stewardship toolkit survey

**DOI:** 10.1186/s13756-025-01525-6

**Published:** 2025-02-21

**Authors:** Yara Mohsen Abd El Azeem Khalaf, Zahira Metwally Gad, Mostafa Ahmed Arafa, Marwa Shawky Abdou

**Affiliations:** Department of Epidemiology, High Institute of Public Health, Alexandria University, Alexandria, Egypt

**Keywords:** Awareness, Perception, Practices, Antimicrobial stewardship implementation program

## Abstract

**Background:**

Antimicrobial resistance (AMR) has been recognized by international policymakers as a serious threat due to its current and projected influence on global population health, healthcare expenditures and gross domestic product (GDP). The present work aimed to assess the awareness, perceptions, and practices of healthcare professionals regarding the implementation of the Antimicrobial Stewardship program (ASP).

**Methods:**

A cross-sectional study was conducted among 310 health care professionals. A self-administered questionnaire based on Antimicrobial Stewardship Toolkit for Acute and Long-Term Care Facilities; Greater New York Hospital Association (GNYAHA) was used to assess perceptions and knowledge about AMR and automatic selective perception.

**Results:**

A total of 310 healthcare professionals participated in the study, of which 60.6% were females, with a mean age of 37.32 ± 8.71, mean years of working of 6.0 ± 5.95 and mean years of experience of 7.97 ± 7.14. The mean scores for AMR awareness, antibiotic (AB) prescribing practices, ASP perception, and intervention beliefs were 73.05 ± 11.21, 32.97 ± 8.22, 52.85 ± 13.99, and 18.24 ± 2.71, respectively. Bivariate analysis showed that profession, primary work unit, staff position significantly affect the AMR awareness and ASP perception, while only staff position affect intervention beliefs. The Multivariate linear regression showed that working in pharmacy, ABs prescribing practice and ASP perception were independent predictors of AMR awareness. Years of work in hospital, AMR awareness and ASP perception were the independent predictors of ABs prescribing practice. AMR awareness, ABs prescribing practice and intervention beliefs were independent predictors of ASP perception while ASP perception was the only independent predictors of intervention beliefs.

**Conclusions:**

The study found a high level of awareness and practice regarding AMR and ASP among healthcare professionals. Pharmacists had higher levels of AMR awareness and ASP perception than physician in different specialities. Levels of AMR awareness, ABs prescribing practice, ASP perception and intervention beliefs affect each other in different ways. Linear regression supported our findings that pharmacists had a higher level of AMR awareness, while years of work in hospital had a significant effect on ABs prescribing practice.

## Introduction

Antimicrobial resistance (AMR) occurs when bacteria, viruses, fungi and parasites are no longer responding to medications making infections harder to treat and increase the risk of disease spread, severe illness and death [1]. 

Despite the fact that AMR has long been recognized as a global problem, there is a significant paucity of data on the patterns and distribution of resistant infections, particularly in developing nations and the Arab world [2]. There is a large clinical and public health burden associated with AMR. It is challenging to estimate the associated morbidity and mortality, particularly in low- and middle-income countries and for community-acquired diseases [3]. The World Bank assessed the economic burden of AMR finding that AMR would elevate the rate of poverty and impact low-income countries compared to the rest of the world [4]. 

Unnecessary and increased antimicrobial use is linked to the development of AMR, not only in individual patients, but also in communities, and regions, posing a risk to individual patients [5]. In Egypt, a study was conducted in 2021, revealed that Egyptian prescribers have a high level of knowledge regarding antibiotics, but they have low level of positive attitude and proper practice when it comes to the problem of AMR [6]. This calls for the importance of Antimicrobial Stewardship program (ASP) implementation on a national level. ASP has shown to reduce the emergence of AMR and health-care-associated infections (HCAIs) and save health-care costs associated with inappropriate antimicrobial use [7]. Though ASP has proven to improve antibiotic use in developed countries, ASP strategies to contain AMR are unsuccessfully executed in developing countries prompting a need to evaluate, detecting gaps, downsides, and physicians’ practices for an effective ASP [8]. 

The present work aimed to assess awareness, perception and practices of healthcare professionals regarding Antimicrobial Stewardship implementation program to gain information about factors affecting the implementation of ASP within Egyptian hospitals, and describe the gaps and limitations of current program.

## Methods

A cross-sectional study was conducted in General Hospitals of Ministry of Health and Population (MoHP), Cairo, Egypt, during the period August 2022 through August 2023 where physicians and clinical pharmacists working in such hospitals were invited to participate after describing the objectives and rationale of the study. The sample size was calculated using Epi Info version 7.2.4.0 (2020). Based on the refusal of health care professional to ASP guiding their antimicrobial prescribing decision and reduction of antibiotic use of 24%, and a margin of error of 5%, the minimum required sample at 95% confidence level was calculated to be 280 health care professional (physicians and clinical pharmacists) and it was rounded to 310. The required sample size was obtained through multistage stratified sampling. First, three medical zones were selected randomly from the five medical zones of Cairo. Then, from each of the selected medical zones, four MoHP hospitals were randomly selected. Finally, in each hospital, physicians and clinical pharmacists were proportionally allocated according to their total number. The health care professionals were consecutively recruited till reaching the required sample size.

### Questionnaire development and data collection

A self-administered questionnaire based on Antimicrobial Stewardship Toolkit for Acute and Long-Term Care Facilities; Greater New York Hospital Association (GNYHA) was used. It is composed of 5 sections: section A contains 22 questions covering the scope of AMR problem and key contributors, section B is formed of 11 questions covering the communication with the microbiology lab, antibiotic susceptibility patterns and antibiotic restriction, section C is constituted of 15 questions covering the role of ASP in improving quality of patient care, impact on hospitals’ nosocomial infection rates, capacity to establish and implement an effective ASP, presence of infectious disease experts that can provide guidance in antibiotic selection and prescription, and staff education regarding antibiotic prescribing and use in hospitals, section D is formed of 7 questions covering the perceptions and beliefs on suggested potential solutions for preventing antimicrobial resistance and the last section was about the socio-economic and work data of the participants. Section E is formed of 8 questions covering the sociodemographic and working characteristics [age, gender, speciality, position, years of experience, years of working inside the hospital, number of patients treated per week and number of antibiotics prescribed per weeks (for physicians only)]. The overall reliability of questionnaire was 0.876. As for the subsections; the Cronbach alpha were 0.713, 0.739, 0.896 and 0.776 for sections A, B, C and D, respectively.

### Data scoring

Scoring sections (A-C) was done using 5-Point Likert Scale, from 1 (Strongly disagree) to 5 (strongly agree). Section D was based on level on agreement where: 1 = probably or definitively ineffective (Not useful); 2 = Unsure; 3 = probably or definitively effective.

### Statistical analysis

The collected data were revised, coded, and analysed the SPSS software (Armonk, NY: IBM Corp version 25.0). The quantitative variables were expressed using mean ± SD while categorical variables were described by counts (%). The mean% was calculated using the following formula: mean score/ total score x 100. The mean% indicate the ratio between the numbers of correctly answered items and the total score of these items. Independent t test and One-way ANOVA test were used to detect any statistically significant difference between continuous variables. Linear regression models using the significant variables were conducted to estimate the significant predictors affecting the AMR awareness, ABs prescribing, ASP perception and intervention beliefs. Statistical significance was considered when *p* < 0.05.

## Results

The total number of participants in the current study were 310, 262 physicians (84.5%) and 48 pharmacists (15.5%). Their mean age was 37.32 ± 8.71 years. 122, (39.4%) were male and 60.6% (188) were female. The primary work areas for the participants were categorized as medicine, non-surgical (118,38.1%), 18.7% (58) in paediatrics, 15.5% (48) in pharmacy, 12.9% (40) in surgery and anaesthesia units, 5.8% [18] in ICU, 5.2% [16] in gynaecology/obstetrics and 4.2% [12] in emergency unit. 30.6% (95) of staff position in the hospital was consultant physicians or attending staff, 20.3% (63) were fellow physicians, 24.5% (76) were resident physicians, 15.5% (48) were pharmacists and 9.0% (28) had other position. Nearly half of them (50.6%; 157) worked in the hospital for less than 5 years, 26.1% (81) participants worked for 5 to less than 10 years, 11.7% (61) participants worked for 10 to less than 20 years and only 3.5% [11] participants worked for more than 20 years in the hospital with mean years of working for all participants of 6.0 ± 5.95 years. Most of (62.9%; 195) participants had less than 10 years of experience in their specialty, 30.6% (95) of them had between 10 and 20 years of experience and 6.5% [20] of them had experience of more than 20 years in their speciality with mean years of experience of 7.97 ± 7.14 years, Table 1.

**Table 1:** Sociodemographic characteristics of the study sample

	Study sample (*n* = 310)
**Age**	
Min. – Max.	22.0–62.0
Mean ± SD	37.32 ± 8.71
**Sex**	
Male	122 (39.4%)
Females	188 (60.6%)
**Profession**	
Physician	262 (84.5)
Pharmacist	48 (15.5)
**Primary work area or unit**	
Medicine (non-surgical)	118 (38.1)
Pediatrics	58 (18.7)
Pharmacy	48 (15.5)
Surgery and anesthesia	40 (12.9)
ICU	18 (5.8)
Gynecology/obstetrics	16 (5.2)
Emergency	12 (4.2)
**Staff position in the hospital**	
Consultant physician or attending staff	95 (30.6)
Fellow physician	63 (20.3)
Resident physician	76 (24.5)
Pharmacist	48 (15.5)
Other	28 (9.0)
**Years of work in hospital**	
<5	157 (50.6)
5- <10	81 (26.1)
10- <20	61 (11.7)
≥20	11 (3.5)
Min. – Max.	1/12–33.0
Mean ± SD	6.0 ± 5.95
**Years of experience in specialty**	
<10	195 (62.9)
10–20	95 (30.6)
>20	20 (6.5)
Min. – Max.	2/12–36 9/12
Mean ± SD	7.97 ± 7.14
**Treated patients per week** ^**a**^	
Min. – Max.	2.0–750.0
Mean ± SD	92.02 ± 135.05
**Treated patients with antibiotics per week** ^**a**^	
Min. – Max.	1.0–500.0
Mean ± SD	47.1 ± 92.7

Data are presented as mean ± SD or frequency (%). ICU: intensive care unit. a: Respondents were only physicians

Table 2 shows the scores of the awareness, perceptions and beliefs of the study participants. The mean score of awareness regarding AMR and contributing factors was 73.05 ± 11.21 (66.4%), while the mean of antibiotic prescribing practices score was 32.97 ± 8.2 (59.9%). The mean perception regarding ASP score was 52.85 ± 13.99 with a mean% of 66.1% and the mean beliefs on potential intervention score was 18.24 ± 2.71 (86.9%).

**Table 2:** Awareness, perception and beliefs of the study sample

Total score	Study sample (*n* = 310)
**Awareness regarding AMR and contributing factors (A)**	
Min. – Max.	33.0–110.0
Mean ± SD	73.05 ± 11.21
Mean% ^a^	66.4%
**Antibiotic prescribing practices score (B)**	
Min. – Max.	0.0–55.0
Mean ± SD	32.97 ± 8.22
Mean% ^a^	59.9%
**Perception regarding ASP score (C)**	
Min. – Max.	0.0–80.0
Mean ± SD	52.85 ± 13.99
Mean% ^a^	66.1%
**Beliefs on potential intervention score (D)**	
Min. – Max.	7.0–21.0
Mean ± SD	18.24 ± 2.71
Mean% ^a^	86.9%

^a^; mean%= mean score/ total score x 100. The mean% indicate the ratio between the numbers of correctly answered items and the total score of these items

**Fig. 1:**
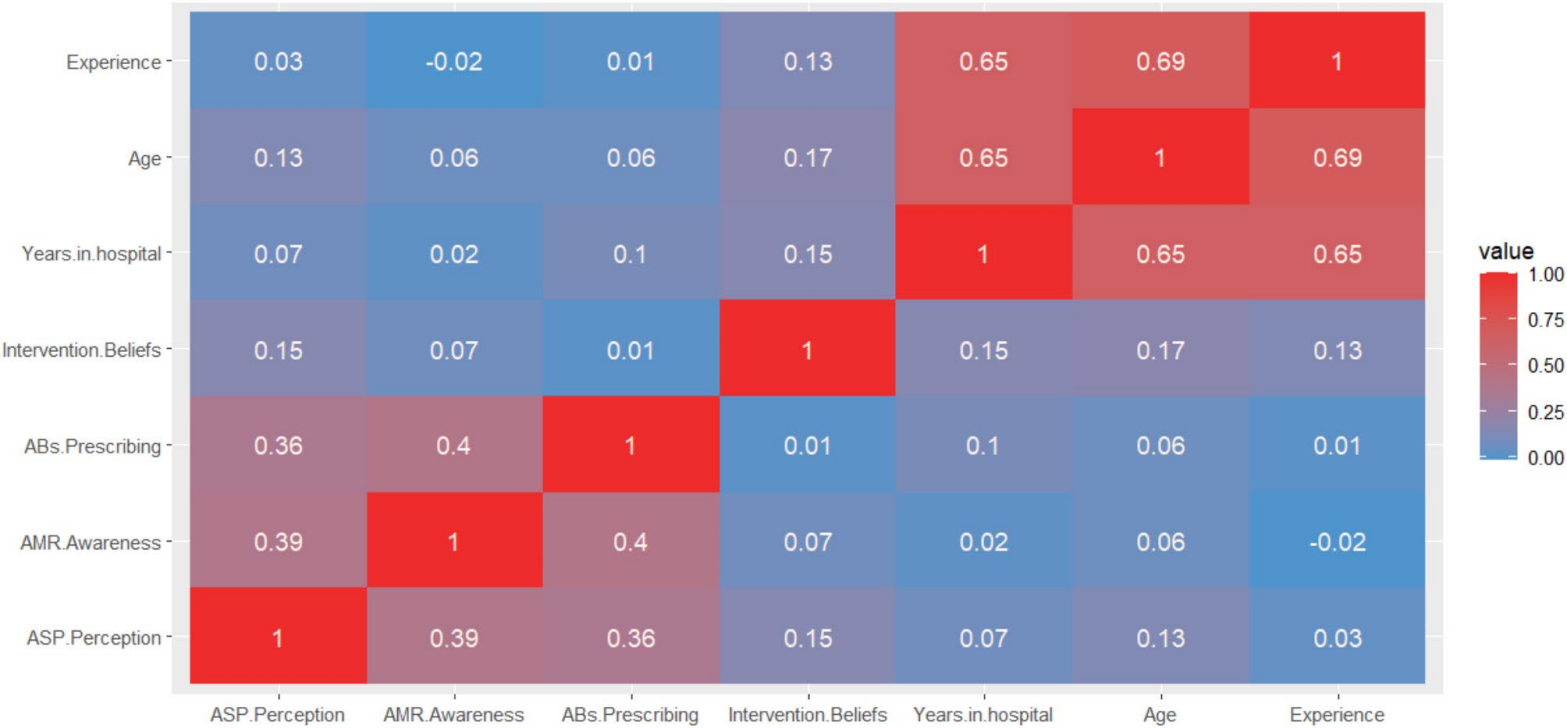
Correlation heatmap

Table 3 describes the distribution of AMR awareness, ABs prescribing practices, ASP perception and intervention beliefs across the different attributes. The mean score for AMR awareness and ASP perception was significantly higher for pharmacists than physicians (80.02 ± 9.83 versus 71.77 ± 10.99, *p* < 0.001 and 32.71 ± 7.79 versus 58.98 ± 8.35, *p* = 0.001, respectively). No significant difference was detected for the four domains with regards to gender. Among physicians, the mean score for AMR awareness was higher significantly for gynecologists (76.63 ± 9.77), followed by ICU physicians (73.11 ± 9.54) and pediatricians (72.76 ± 10.1), while for ASP perception, it was significantly higher among ICU physicians (58.06 ± 7.27) followed by gynecologists (54.69 ± 19.08). Consultants had the highest mean score for the AMR awareness (71.75 ± 11.83), ASP perception (52.72 ± 16.83) and intervention beliefs (18.61 ± 2.71) domains, while fellow physicians had the highest mean score for ABs prescribing domain (33.79 ± 8.14). With regards to duration of work in the hospital and years of experience, no significant difference was detected for the four domains (*p* > 0.05). As for physicians, number of treated patients was found to be positively correlated with their AB prescribing practice and intervention beliefs. Figure 1 shows the correlation heatmap of the studied four scores.

**Table 3:** Factors affecting awareness, perception and beliefs of the study sample

	AMR Awareness	*p*	ABs Prescribing	*p*	ASP Perception	*p*	Intervention Beliefs	*p*
**Sex**		0.371		0.306		0.755		0.806
Male	72.34 ± 12.28		33.57 ± 8.14		53.16 ± 13.53		18.19 ± 2.70	
Female	73.51 ± 10.46		32.59 ± 8.27		52.65 ± 14.31		18.27 ± 2.72	
**Profession**		< 0.001*		0.192		0.001*		0.230
Physicians	71.77 ± 10.99		32.71 ± 7.79		51.73 ± 14.53		18.16 ± 2.80	
Pharmacists	80.02 ± 9.83		34.4 ± 10.23		58.98 ± 8.35		18.67 ± 2.11	
**Primary work area or unit**		0.001*		0.517		< 0.001*		0.408
Medicine (non-surgical)	71.03 ± 11.54^#^		32.42 ± 7.77		51.86 ± 15.0^#^		18.02 ± 2.85	
Pediatrics	72.76 ± 10.1^#^		31.63 ± 8.65		50.37 ± 13.55^#^		18.49 ± 2.65	
Pharmacy	80.02 ± 9.83		34.4 ± 10.23		58.98 ± 8.35^$&^		18.67 ± 2.11	
Surgery and anesthesia	70.1 ± 12.37^#^		33.35 ± 7.51		49.2 ± 14.63^#^		17.45 ± 3.4	
ICU	73.11 ± 9.54^#^		34.56 ± 6.43		58.06 ± 7.27^$&^		18.11 ± 2.45	
Gynecology/obstetrics	76.63 ± 9.77		35.13 ± 7.16		54.69 ± 19.08^$&^		19.19 ± 1.68	
Emergency	71.5 ± 6.46^#^		32.67 ± 6.79		51.83 ± 14.36		19.08 ± 1.83	
**Staff position in the hospital**		< 0.001*		0.481		0.001*		0.027*
Consultant physician/ attending staff	71.75 ± 11.83^#^		32.73 ± 8.63		52.72 ± 16.83^+^		18.61 ± 2.71^+@^	
Fellow physician	71.44 ± 10.72^#^		33.79 ± 8.14		51.6 ± 15.0^#^		18.38 ± 2.24	
Resident physician	71.53 ± 11.49^#^		30.04 ± 6.72		51.24 ± 12.62^#^		17.78 ± 3.14	
Pharmacist	80.02 ± 9.83		34.4 ± 10.23		58.98 ± 8.35^+^		18.67 ± 2.11^+^	
Other	73.21 ± 6.91^#^		32.04 ± 6.69		50.04 ± 9.37^#^		17.14 ± 2.97	
**Years of work in hospital**		0.785		0.627		0.181		0.233
<5	72.78 ± 12.36		32.75 ± 8.26		52.08 ± 14.1		17.88 ± 2.97	
5-<10	73.49 ± 9.3		32.43 ± 8.1		54.0 ± 12.36		18.37 ± 2.67	
10-<20	73.33 ± 10.27		33.79 ± 8.2		52.05 ± 16.29		18.87 ± 1.94	
≥20	72.0 ± 13.05		35.64 ± 8.89		60.0 ± 7.24		18.82 ± 1.99	
**Years of experience in specialty**		0.201		0.311		0.059		0.057
<10	73.61 ± 11.05		33.19 ± 8.04		53.09 ± 12.47		17.94 ± 2.87	
10–20	71.55 ± 11.34		32.06 ± 8.13		50.92 ± 17.2		18.75 ± 2.41	
>20	74.65 ± 12.01		35.1 ± 10.11		59.75 ± 7.94		18.65 ± 1.87	
**Treated patients per week**	-0.036	0.561	0.136	0.027*	0.056	0.367	0.188	0.002*
**Treated patients with ABs per week**	-0.028	0.651	0.117	0.06	0.071	0.252	0.167	0.007*

*; Significant (*p* < 0.05), #; Significant with pharmacy/pharmacist, $; Significant with surgery and anesthesia, &; Significant with Pediatrics, +; significant with other, @; significant with resident physician. Antimicrobial resistance (AMR)

The multivariate linear regression analysis showed that the significant predictors affecting AMR awareness score were primary work area (pharmacy) *p* < 0.001, ABs prescribing practice score, *p* < 0.001 and ASP perception, *p* < *0.001*. The model explains 27.9% of variability, where R^2^ was 27.9%. The significant predictors affecting ABs prescribing practice score were years of work in hospitals, *p* = 0.034, AMR awareness score, *p* < 0.001 and ASP perception score, *p* < 0.001. The model explains 23.1% of variability in the dependent variable, where R^2^ was 23.1%. Significant predictors affecting ASP perception score were AMR awareness score, *p* < 0.001, ABs prescribing practice score, *p* < 0.001 and intervention beliefs, *p* = 0.041, R^2^ was 24.6%. Only one significant predictor affecting intervention beliefs, which was ASP perception score. Table 4.

**Table 4:** Multivariate analysis to assess the independent contribution of different factors affecting awareness, perception, practice and beliefs

Predictors	Unstandardized Coefficients		Standardized Coefficients	t	Individual Predictors Sig	95% Confidence Interval
	B	Std. Error	Beta			
**AMR awareness** ^#^						
**Constant**	45.268	5.131		8.823	< 0.001*	35.171–55.365
**Age**	0.068	0.096	0.053	0.710	0.478	-0.121–0.258
**Primary work area or unit**						
Pediatrics	2.420	1.558	0.085	1.554	0.121	-0.645–5.486
Pharmacy	6.916	1.748	0.222	3.957	< 0.001*	3.476–10.355
Surgery and anesthesia	-0.669	1.793	-0.020	-0.373	0.709	-4.197–2.859
ICU	-0.036	2.479	-0.001	-0.014	0.989	-4.915–4.844
Gynecology/obstetrics	3.866	2.628	0.076	1.471	0.142	-1.305–9.037
Emergency	0.407	2.952	0.007	0.138	0.890	-5.403–6.217
**Years of work in hospital**	-0.096	0.134	-0.051	-0.719	0.473	-0.360–0.167
**Years of experience in specialty**	0.007	0.120	0.004	0.055	0.956	-0.229–0.243
**ABs prescribing practice**	0.408	0.073	0.299	5.598	< 0.001*	0.265–0.551
**ASP perception**	0.191	0.044	0.238	4.330	< 0.001*	0.104–0.278
**Intervention beliefs**	0.032	0.212	0.008	0.149	0.882	-0.386–0.449
**ABs prescribing practice** ^**$**^						
**Constant**	11.456	4.315		2.655	0.008*	2.965–19.947
**Age**	-0.019	0.073	-0.021	-0.266	0.790	-0.163–0.124
**Primary work area or unit**						
Pediatrics	-1.108	1.183	-0.053	-0.936	0.350	-3.436–1.220
Pharmacy	-1.155	1.356	-0.050	-0.851	0.395	-3.824–1.515
Surgery and anesthesia	1.456	1.355	0.059	1.074	0.284	-1.211–4.123
ICU	1.074	1.877	0.031	0.572	0.568	-2.619–4.768
Gynecology/obstetrics	1.504	1.996	0.041	0.754	0.452	-2.423–5.431
Emergency	0.071	2.236	0.002	0.032	0.975	-4.329–4.472
**Years of work in hospital**	0.215	0.101	0.156	2.135	0.034*	0.017–0.413
**Years of experience in specialty**	-0.100	0.091	-0.087	-1.102	0.271	-0.278–0.079
**AMR awareness**	0.234	0.042	0.319	5.598	< 0.001*	0.152–0.316
**ASP perception**	0.141	0.033	0.241	4.224	< 0.001*	0.075–0.207
**Intervention beliefs**	-0.151	0.161	-0.050	-0.943	0.346	-0.467–0.164
**ASP perception** ^**&**^						
**Constant**	-0.165	7.357		-0.022	0.982	-14.642–14.313
**Age**	0.203	0.122	0.126	1.658	0.098	-0.038–0.443
**Primary work area or unit**						
Pediatrics	-1.957	1.993	-0.055	-0.982	0.327	-5.879–1.966
Pharmacy	3.600	2.279	0.092	1.580	0.115	-0.885–8.085
Surgery and anesthesia	-2.047	2.285	-0.049	-0.896	0.371	-6.544–2.451
ICU	4.842	3.152	0.081	1.536	0.126	-1.361–11.045
Gynecology/obstetrics	-0.480	3.366	-0.008	-0.143	0.887	-7.104–6.144
Emergency	-1.017	3.768	-0.014	-0.270	0.787	-8.432–6.398
**Years of work in hospital**	-0.016	0.171	-0.007	-0.095	0.925	-0.353–0.320
**Years of experience in specialty**	-0.077	0.153	-0.039	-0.502	0.616	-0.378–0.224
**AMR awareness**	0.311	0.072	0.249	4.330	< 0.001*	0.170–0.452
**ABs prescribing practice**	0.401	0.095	0.236	4.224	< 0.001*	0.214–0.588
**Intervention beliefs**	0.553	0.269	0.107	2.057	0.041*	0.024–1.083
**Intervention beliefs** ^**@**^						
**Constant**	15.793	1.282		12.320	< 0.001*	13.270–18.315
**Age**	0.029	0.026	0.095	1.122	0.263	-0.022–0.081
**Primary work area or unit**						
Pediatrics	0.455	0.427	0.066	1.067	0.287	-0.385–1.295
Pharmacy	0.623	0.489	0.083	1.274	0.204	-0.339–1.585
Surgery and anesthesia	-0.415	0.490	-0.052	-0.848	0.397	-1.379–0.548
ICU	0.073	0.678	0.006	0.107	0.915	-1.261–1.407
Gynecology/obstetrics	1.131	0.718	0.093	1.576	0.116	-0.282–2.544
Emergency	0.987	0.805	0.070	1.226	0.221	-0.597–2.571
**Years of work in hospital**	0.036	0.037	0.078	0.973	0.331	-0.036–0.108
**Years of experience in specialty**	0.005	0.033	0.012	0.143	0.887	-0.060–0.069
**AMR awareness**	0.002	0.016	0.010	0.149	0.882	-0.029–0.034
**ABs prescribing practice**	-0.020	0.021	-0.060	-0.943	0.346	-0.061–0.021
**ASP perception**	0.025	0.012	0.131	2.057	0.041*	0.001–0.050

^#^; F = 9.600, *p* < 0.001*, R^2^ = 27.9%, ^$^; F = 7.423, *p* < 0.001*, R^2^ = 23.1%, ^&^; F = 8.094, *p* < 0.001*, R^2^ = 24.6%, ^@^; F = 2.016, *p* = 0.023, R^2^ = 7.5%, Linear regression equations for significant predictors are

AMR awareness = 45.268 + 6.916 (Pharmacy) + 0.408 (ABs prescribing practice) + 0.191 (ASP perception)

ABs prescribing practice = 11.456 + 0.215 (years of work in hospital) + 0.234 (AMR awareness) + 0.141 (ASP perception)

ASP perception= -0.165 + 0.311 (AMR awareness) + 0.401 (ABs prescribing practice) + 0.553 (Intervention beliefs)

Intervention beliefs = 15.793 + 0.025 (ASP perception)

## Discussion

Antimicrobial resistance is a significant problem in Egyptian hospitals. A recent study revealed that out of a total of 20,353 isolates, 9,751 (48%) were found to be positive for AMR [9]. ASP programs are the most effective programs that aim to optimize patient safety, quality of care and minimize AMR. In addition, they significantly contribute to the healthcare system through promoting and monitoring antimicrobial agents [10]. 

The current study revealed different mean total scores for AMR awareness and contributing factors score was 73.05 ± 11.21, the antibiotic prescribing practices, 32.97 ± 8.22, ASP perception, 52.85 ± 13.99 and beliefs on potential intervention, 18.24 ± 2.71.

Mittal et al., [11] demonstrated that an overwhelmingly high proportion of the respondents believed indiscriminate use of antibiotics (98.2%) and use of broad-spectrum agents (95.8%) contribute to AMR. Most of the participants (76.8%) believed that their antibiotic prescribing behavior has an impact on the development of antibiotic resistance in their region. Most of their participants showed positive practice regarding the strategies that can be helpful in handling the issue of AMR with a mean of 15.3 as antibiotic use restricted to cases with confirmed bacterial infections was in 69% of participants and reduced non-prescription sale of antibiotics in 80%, while Hayat et al., [12] illustrated that most of their participants viewed AMR to be a serious problem. However, Sefah et al., [13] demonstrated that majority of participants had poor knowledge and poor practice but a good attitude towards AMS.

Our results revealed that AMR awareness was significantly higher among pharmacists compared to physicians, particularly in primary work areas and staff positions such as consultant physicians. In consistence to the results, Tripathi et al. [14] and Hayat et al. [12] found that AMR awareness showed a significant different among different professionals being higher in pharmacists than medical doctor as physicians were less likely to consider AMR as a serious threat in their practicing hospitals compared to pharmacists. Sefah et al. [13] reported different predictors for AMS as sex and continuous professional development training on AMS in the previous years.

According to our results, ASP perception was significantly higher among pharmacists than physicians. ASP perception showed also significant results among the primary work unit in hospital and among staff position in the hospital. This came in line with Mittal et al., [11] who illustrated that specialists/super-specialists from basic and medicine/allied sciences were found to be associated with higher scores of knowledges, attitudes and practices (KAP) in comparison to non-specialists. Working in secondary healthcare settings was significantly associated with lower scores as compared to tertiary care. Other factors such as age, gender, years of practice, and highest educational qualification were not found to have an influence on aggregate KAP scores among participants. In contrast, Sefah et al. [13] reported that ASP knowledge score was insignificantly different between pharmacist and medical doctors. But they showed that attitude scores were significantly higher in pharmacist than medical doctors. Hayat et al. [12] found that attitude of healthcare professionals towards strategies of hospital ASPs was significantly different among different professionals being higher in physicians. The different culture in Pakistan and different sample size in their population may explain this difference from our results.

In the current study, intervention beliefs were insignificantly different in sex, profession, primary work area, years of work in hospital and years of experience in specialty were insignificantly different among all groups. Staff position in the hospital was significantly higher in (consultant physician/ attending staff and pharmacist) than other. There was a positive correlation between (treated patients per week and treated patients with ABs per week) and intervention beliefs. Hayat et al. [12] illustrated that restriction of prescription of certain antibiotic was significantly different between different professionals. Also, Sefah et al. [13] found that the level of practice of AMS among healthcare professionals was associated with the profession but they found that AMS was associated with their duration of experience. Moreover, Tripathi et al. [14] found that intervention beliefs were significantly different among different professionals being higher in pharmacists than medical doctor.

Also, positive correlation was found of treating patients per week and AB prescribing practice and intervention beliefs among physicians. Treating more patients enhances physicians’ experience, which in turn improves their perceptions and beliefs regarding antibiotics, AMR, and ASP.

The linear regression of the work illustrated that the primary work area (pharmacy), ABs prescribing practice and ASP perception were the significant independent predictors of AMR awareness. Simegn et al. [15] who carried out a cross-sectional survey among health professionals in Ethiopia found that the use of antibiotics practice was associated with knowledge of AMR. Furthermore, Karasneh et al. [16] conducted a survey among Jordanian physicians and dentists and demonstrated that profession was a significant predictor of knowledge to prescribe antibiotics.

The findings also revealed that years of work in hospital, AMR awareness and ASP perception were the significant independent predictors of ABs prescribing practice. Higuita-Gutiérrez et al. [17] carried out a descriptive study to assess knowledge, attitude, and practice regarding antibiotic use and resistance among medical students in Colombia. They found that ABs prescribing practice was associated with AMR awareness. Also, Anong and Akoachere [18] conducted a retrospective study to assess prescribing patterns and associated factors of antibiotic prescription in Cameroon and found that years of work experience was an independent predictor of ABs prescription.

AMR awareness, ABs prescribing practice and intervention beliefs were independent predictors of ASP perception. Sefah et al. [13] illustrated that the exposure to ASP structured training was independent predictor of ASP knowledge and continuous professional development training on ASP last year while age and profession were not. They also found that there was a statistically significant difference in the mean score of the attitude and practice of AMS among the healthcare professionals. Moreover, Tegagn et al. [19] conducted a study to evaluate knowledge, attitudes and practices of healthcare professionals towards antimicrobial stewardship and their predictors in Ethiopia. They found that age, profession and years of experience were not significant predictors of health care professionals towards antibiotic stewardship. On contrast, they found that there was no relation between knowledge and practice towards antibiotic stewardship.

### Limitations of the study

The present work was a descriptive cross sectional self-administered study that faced a reluctant in the response of the healthcare professionals during their working time in the hospitals which might affect the significance of their responses. The study was conducted in only one governorate out of 29 governorates in Egypt which affect the generalizability of the results. Also, nurse professions, dentists and other healthcare providers were not included in the study which leave limited knowledge about their awareness, perceptions and beliefs regarding ASP implementation.

## Conclusions

The work showed that healthcare professional had a good level of awareness and practice regarding AMR and ASP. Pharmacists demonstrated higher levels of AMR awareness and ASP perception compared to physicians across various specialties. levels of AMR awareness, ABs prescribing practice, ASP perception and intervention beliefs affect each other in different ways. Linear regression supported the findings that pharmacists are significantly had a higher level of AMR awareness, while years of work in hospital had a significant effect on ABs prescribing practice.

## Data Availability

No datasets were generated or analysed during the current study.
